# Is Carbon Dioxide (CO_2_) Emission an Important Factor Affecting Healthcare Expenditure? Evidence from China, 2005–2016

**DOI:** 10.3390/ijerph16203995

**Published:** 2019-10-18

**Authors:** Linhong Chen, Yue Zhuo, Zhiming Xu, Xiaocang Xu, Xin Gao

**Affiliations:** 1 School of Public Administration, Sichuan University, Chengdu 610065, China; 2017325010005@stu.scu.edu.cn; 2 School of Mathematics and Statistics, Chongqing Technology and Business University, Chongqing 400067, China; 3 Finance office, Sichuan University, Chengdu 610065, China; 4 Department of Business, ESCP Europe Business School, 75011 Paris, France; tiantian20120424@126.com; 5 School of Economics, Chongqing Technology and Business University, Chongqing 400067, China; 6 Business School, Hohai University, Nanjing 211100, China; gxtz1987@hhu.edu.cn

**Keywords:** Carbon dioxide (CO_2_) emission, Income, Health care expenditure (HCE), Government financial expenditure, Bayesian quantile regression (BQR), Traditional empirical methods

## Abstract

As a result of China’s economic growth, air pollution, including carbon dioxide (CO_2_) emission, has caused serious health problems and accompanying heavy economic burdens on healthcare. Therefore, the effect of carbon dioxide emission on healthcare expenditure (HCE) has attracted the interest of many researchers, most of which have adopted traditional empirical methods, such as ordinary least squares (OLS) or quantile regression (QR), to analyze the issue. This paper, however, attempts to introduce Bayesian quantile regression (BQR) to discuss the relationship between carbon dioxide emission and HCE, based on the longitudinal data of 30 provinces in China (2005–2016). It was found that carbon dioxide emission is, indeed, an important factor affecting healthcare expenditure in China, although its influence is not as great as the income variable. It was also revealed that the effect of carbon dioxide emission on HCE at a higher quantile was much smaller, which indicates that most people are not paying sufficient attention to the correlation between air pollution and healthcare. This study also proves the applicability of Bayesian quantile regression and its ability to offer more valuable information, as compared to traditional empirical tools, thus expanding and deepening research capabilities on the topic.

## 1. Introduction

It is widely believed that economic growth and population health have significantly improved in China over the past few decades, with a sharp fall in mortality rates, increased life expectancy, and expansion of immunization coverage. In 2016, the infant mortality rate in the country was 7.5‰, a decrease by 76.7% from 2000. However, the increasing air pollution, including carbon dioxide (CO_2_) emission, is causing grievous health problems and accompanying heavy economic burdens on healthcare [1,2,3,4]. According to The China Statistical Yearbook (2017), carbon dioxide (CO_2_) emissions per capita increased to 5.5 tons in 2016 from 3.6 tons in 2004. The number of disabled and semi-disabled elderly was 40.63 million in China, accounting for 18.3% of the elderly population [5].

The effect of air pollution on physical health has been confirmed by several scholars in the fields of sociology, economics and medicine. Xie et al. proved a linkage between fine particulate emission and ischaemic heart disease mortality and morbidity, based on time series data on Beijing, China [6]. Spix &Wichmann found that CO_2_ caused a 3–4% growth in mortality rate and twice as high caused by particulates in Koln Germany [7]. Similar studies are abundant in other countries, such as the US [8] and India [9].

With Ridker [10] initiating discussions on the economic costs of air pollution, several studies on the relationship between air pollution and healthcare expenditure (HCE) have emerged across the globe. Almost all of the studies have confirmed that air pollution, especially CO_2_, has a positive effect on healthcare expenditure (HCE) [11,12,13,14,15,16], which is also consistent with the common man’s basic understanding. Further, some scholars have introduced economic factors (such as income) and formed a discussion of the tripartite relationship between CO_2_ emissions, economic factors, and healthcare expenditure (HCE). For example, Chaabouni, S. & Saidi, K. discussed the multilateral relationship between CO_2_ emissions, healthcare expenditure, and GDP (Gross Domestic Product) in 51 countries during 1995–2013, and found that there is a unidirectional causality from CO_2_ emissions to healthcare expenditure, except in low-income countries [17]. Yazdi, S.K. & Khanalizadeh, B. show that income elasticity is inelastic—health expenditure is not more sensitive to income and adjustment to changes in income in MENA countries [18]. In recent years, there have been a large number of studies on China [19,20,21,22,23,24,25]. For instance, Li et al. [26] calculated the economic burden caused by air pollution (PM_10_ and CO_2_) and showed that the economic loss makes up about 1.63% and 2.32% of the Gross Domestic Product, respectively. 

There are a variety of empirical research tools used in this field. For instance, in addition to panel data analysis used by some scholars [27,28,29], the two-stage time series regression model was used by Jerrett et al. to show a correlation between air pollution and healthcare expenditure (HCE) in Ontario, Canada [30]. Using OLS estimators, Narayan et al. revealed that air pollution, including nitrogen oxide and carbon monoxide emissions, has had a prominent positive influence on healthcare expenditure (HCE) for a long time [31]. Chaabouni adopted the dynamic simultaneous equation model to discuss the correlation between CO_2_ emissions, HCE, and economic development in lower and higher-income countries during 1995–2013 [32]. The quantile regression approach (QR) has also been used by some scholars, such as in a case study by Nicholas et al. on U.S. state-level CO_2_ emissions and healthcare expenditure [33]. 

In summary, as shown in Table 1, academic studies on the relationship between air pollution (especially CO_2_ emission) and healthcare expenditure (HCE) have begun to emerge and related empirical tools, such as the OLS model, are increasingly being used. However, as stated earlier, only a few studies have attempted to adopt the quantile regression model (QR) [33] and the Bayesian quantile regression model (BQR) [34], which have limited characteristics relative to traditional empirical methods. For instance, the assumption of independent or identically distributed errors, which is necessary for ordinary least squares (OLS), is not required in BQR [35]. As a continuation of our previous work [34], this paper attempts to introduce the Bayesian quantile regression model (BQR) to discuss the correlation between CO_2_ emission and healthcare expenditure (HCE) in China. Admittedly, there are several undeveloped aspects that need to be addressed in future research. It should be pointed out that we are not trying to prove its superiority, but emphasize its applicability as a new empirical method that can expand and deepen discussions on the issue.

This paper aims to answer two questions. First, is CO_2_ emissions an important factor affecting healthcare expenditure (HCE) in China? Second, how do CO_2_ emissions affect HCE? The rest of the paper is arranged as follows: Section 2 details the establishment of the BQR model, selection of variables, and data sorting; Section 3 discusses the empirical test and results of the BQR; Section 4 and Section 5 provide a detailed discussion and policy conclusions of the empirical results.

## 2. Materials and Methods 

### 2.1. Estimation Method: Bayesian Quantile Regression Model (BQR)

Since its introduction by Koenker & Basset [36], quantile regression (QR) has become a significant empirical research approach in the fields of sociology and economics, among others. As shown in the R package-quantreg [37], the usual way to derive quantile regression is by the following standard linear model. Consider the classical formula Equation (1) where $x_{i}$ and β are column vectors, and $y_{i}$ is a scalar variable:

(1) $$y_{i}=x_{i}^{˕}\beta +\epsilon_{i}$$

When E(ϵ|x)=0 or Med(ϵ|x)=0, we can obtain its conditional mean model or conditional median model. 

In 2001, Yu & Moyeed [38] used the Bayesian method in quantile regression for the first time. The Bayesian execution of quantile regression begins with the formation of a likelihood of independent ALD (Asymmetric Laplace Distribution) with $\mu =x_{i}^{˕}\beta$. The quantile of interest, τ, has to then be specified and priors added to the model parameters, β and σ. The resulting posterior distribution can be represented as follows:

(2) $$\psi =\left(\beta ,\sigma |y,x,\tau \right)\propto \pi \left(\beta ,\sigma \right)\overset{n}{\underset{i=1}{\prod }}ALD(y_{i}|x_{i}^{˕}\beta ,\sigma ,\tau )$$

where π(β,σ) is the joint prior on the regression parameters, and the inference about model parameters follows conventional Bayesian procedures.

The Bayesian quantile regression model (BQR) is more flexible than traditional regression methods (such as OLS). For example, it is more relaxed about the assumptions on independent and identically distributed error [39]. The BQR theory has seen rapid developments [35,40]; however, it has hardly been used in the literature to discuss the factors influencing healthcare expenditure (HCE), especially the relationship between air pollution and HCE.

The contribution of this paper lies in its attempt to use the Bayesian quantile regression model (BQR) as a newish empirical tool to discuss the correlation between CO_2_ emission and healthcare expenditure (HCE) in China. It is a continuation of our previous work [34]. 

### 2.2. Variables and Data Sources

A Bayesian quantile regression (BQR) model was built to discuss the impact of carbon dioxide (CO_2_) emission on healthcare expenditure (HCE), as shown in Equation (3):

(3) $$\ln(HCE_{t})=\beta_{0}+\beta_{1}\ln\left(INCOME_{t}\right)+\beta_{2}\ln\left(CO2_{t}\right)+\beta_{3}\ln\left(DCLI_{t}\right)+\beta_{4}\ln\left(GFE_{t}\right)\;+\beta_{5}\ln\left(ODR_{t}\right)+\beta_{6}\ln\left(CD_{t}\right)+\beta_{7}\ln\left(HT_{t}\right)+\varepsilon_{t}$$

where *t* represents the time period (2005-2016). 

By referring to recent literature [34,41], we chose healthcare expenditure (HCE) as the dependent variable, and carbon dioxide (CO_2_) emission and income as the core independent variables. Other independent variables include government financial expenditure, chronic disease, density of commercial life insurance, old dependency ratio, and health technician. All independent variables were divided into four categories: (a) environment pollution variables, (b) economic variables, (c) public service variables, and (d) family and personal variables. Similar to [34], the above variables are abbreviated as HCE, CO_2_, INCOME, GFE, DCLI, ODR, CD, and HT, respectively, (as shown in Table 2).

This information was gathered from the longitudinal data of 30 provinces in China from 2005 to 2016; it excludes Tibet, Taiwan, Hong Kong, and Macao. The data on HCE, INCOME, GFE, and ODR were obtained from China’s National Bureau of Statistics (CNBS). The data on CO_2_ came from the China Environmental Statistics Yearbook (CESY); DCLI from the Yearbook of China’s Insurance (CIRY); and CD and HT from the China Health and Family Planning Statistical Yearbook (CHFPSY). 

**Figure 1:**
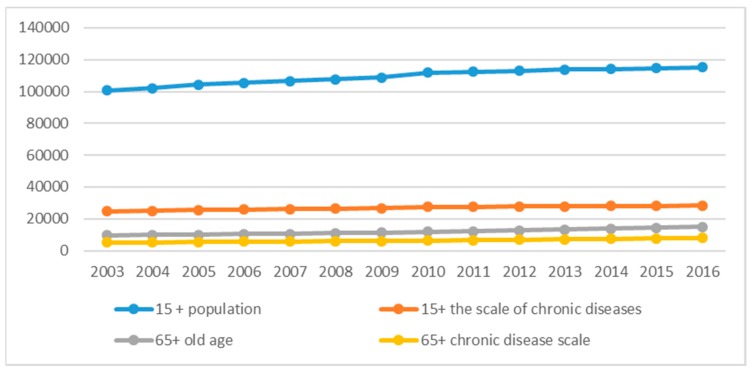
Evolution of chronic diseases in China from 2003 to 2016.

Three things are to be pointed out: First, it is reasonable to use carbon dioxide (CO_2_) emissions as a representation of air pollution, as adopted and proven by several research works. Second, ‘chronic diseases’ in this paper is a hybrid concept, which mean that it does not refer to a specific chronic disease, but to people with at least one. This is consistent with the definition in China Health and Family Planning Statistical Yearbook (CHFPSY). Third, data on chronic diseases need to be measured by their prevalence rate, based on data of 2013 (the latest data available). The prevalence rate has maintained relative stability (538.8‰ in 2003 and 539.9‰ in 2013). Figure 1 shows the trend of chronic diseases in China from 2003 to 2016.

The empirical method followed in this paper is referred to as the R package-bayes QR. All data on price was standardized to the constant price in 2004 and converted to their natural logarithms to decrease their dimensional effects.

## 3. Results

### 3.1. Description of Statistical Characteristics

Table 3 shows the descriptive statistics for each variable.

Table 3 summarises the characteristics of all variables, with Part A denoting data in original value and Part B the adjusted data with log difference. The mean unadjusted HCE was ¥688.32 and INCOME was ¥13,174.28, which implies that people have spent 5.22% of their income on individual healthcare. The mean unadjusted CO_2_ was 4.19(10,000 tons)/10,000 people. 

**Figure 2:**
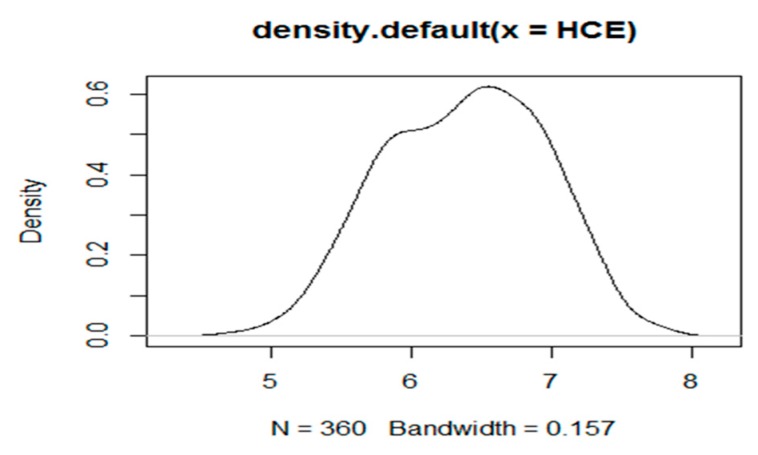
Density function diagram of HCE(Health care expenditure).

The analysis also shows slight skewness for HCE (as shown in Figure 2), which means that the regression results achieved by traditional mean regression models, such as OLS, are practicable, but may have some deviation. Therefore, the regression analysis by the Bayesian quantile regression (BQR) model is considered reasonable, when compared with traditional mean regression models such as OLS.

### 3.2. Empirical Test

#### 3.2.1. Stability Test—ADF (Augmented Dickey-Fuller) Test and Pool Test

In order to check the stationarity of all the variables, especially the dependent HCE, the augmented Dickey-Fuller test (ADF) was conducted. Table 4 reported the results of this test. In addition, the basis for model selection can be obtained through a series of tests, such as the Pool test. 

As demonstrated in Table 3, the augmented Dickey-Fuller test (ADF) provided powerful evidence (at a significance level of 1%) that all variables are stationary, except CD (at a significance level of 10%). These results support the view that a long-term stable correlation exists between variables. Moreover, the Pool test reveals that both individual and time effects (row 5 in Table 3) are not significant. Thus, the mixed model for BQR is a reasonable choice.

#### 3.2.2. Visual Test of MCMC (Markov Chain Monte Carlo) Convergence

The MCMC (Markov Chain Monte Carlo) convergence test is another important analysis to ensure numerical stability for BOR. In this paper, the number of MCMC iterations was set to 5000; the post-acceptance check identifies if this is sufficient to find convergence in the MCMC chain after several BQR experiments. 

**Figure 3:**
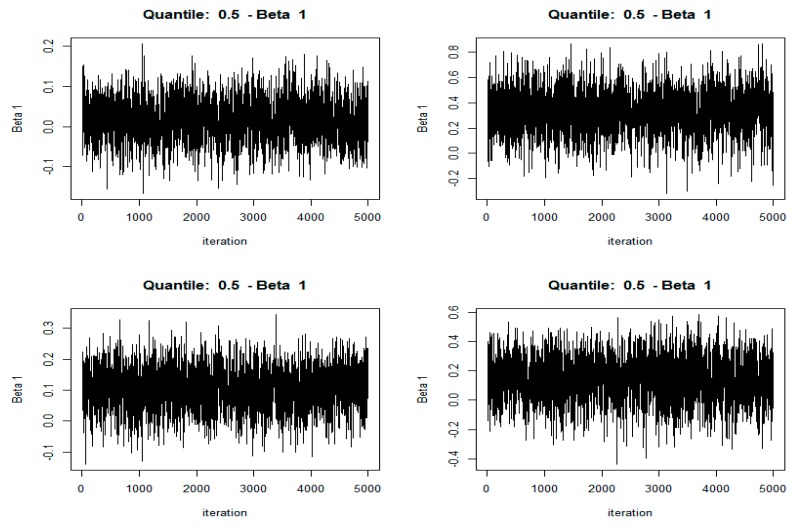
Traceplots of the MCMC (Markov Chain Monte Carlo) chains for intercept (the upper left), HCE (the upper right), CO_2_ (the lower left), and INCOME (the lower right) for BQR.

The visual test of MCMC convergence for Bayesian quantile regression analysis was shown in Figure 3. Only HCE, CO_2_, and INCOME are listed here due to space constraints. The MCMC sampler moves rapidly towards smooth distribution and mixes well, indicating good convergence of the MCMC chain. Here, the display of edge posteriori distribution by rendering the histogram drawn by simulation was omitted. 

### 3.3. Empirical Results of Bayesian Quantile Regression

Table 5 showed the coefficient estimates of Bayesian quantile regression (BQR) in all the quantiles, which is an advantage of quantile regression over OLS. Some interesting findings with respect to the influence of determinants on HCE were revealed in Table 4.

First, the coefficients of INCOME and GFE were large, but not changed much, across all quantiles. For example, the coefficient of INCOME was 0.356 (τ = 0.1), 0.331 (τ = 0.5), and 0.313 (τ = 0.9), respectively; the coefficient of GFE was 0.302 (τ = 0.1), 0.274 (τ = 0.5), and 0.312 (τ = 0.9), respectively; which illustrated that income and government financial expenditure were the most important factors affecting the HCE for all people. 

Second, DCLI and ODR have a special effect on HCE. The coefficient of DCLI was −0.001(τ = 0.1), 0.089 (τ = 0.5), 0.072 (τ = 0.7), and 0.051 (τ = 0.9), which, although increasing, was relatively stable. This implies that people are paying more attention to their own health (life insurance and HCE increase simultaneously), although a substitution relationship between DCLI and HCE (−0.001, τ = 0.1) was found for a small number of people. 

Finally, CO_2_, the focus of our analysis, is an important factor affecting healthcare expenditure (HCE), which can be found by the coefficients of CO_2_ on HCE in all quantiles. The coefficients of CO_2_ were 0.101 (τ = 0.1), 0.157 τ = 0.5), and increased to 0.227 (τ = 0.9), which reveals that CO_2_ emissions have an increasing influence on HCE. However, this effect was still little, compared to INCOME. It was revealed that the majority of people do not pay sufficient attention to the correlation between air pollution and healthcare.

### 3.4. Comparison of Bayesian Quantile Regression (BQR) and the Traditional Empirical Methods

Table 6 shows the estimated results of four methods: OLS, BLR, QR, and BQR. Despite a slight difference in the basic theoretical principles of the Bayes method and the frequency method (such as OLS), possibly due to less precise macro data or no enough sample size adopted in our paper, the results of the BQR and the other methods were quite similar. For example, the coefficients of INCOME were 0.337 (OLS) and 0.331 (BQR). Thus, the rationality of BQR has been tested.

**Figure 4:**
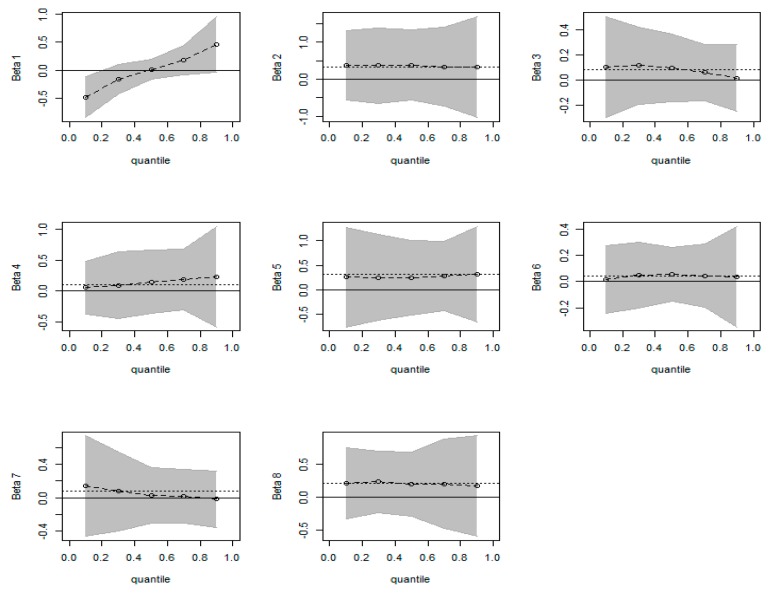
Quantile plots for BQR. The dotted lines represent the OLS estimate. The shaded area shows the adjusted credible intervals as the parameters are estimated. The variables represented by each graph are intercept, INCOME, CD, CO_2_, GFE, DCLI, ODR, and HT, respectively.

Corresponding to Table 6, Figure 4 provides the quantile graphics of all the variables, including intercept for BQR compared to OLS. The full line represents zero, the dotted line represents the estimated outcomes of OLS, and the dashed lines represent the estimates of different quantiles for BQR. The shaded area represents the upper/lower estimated values of BQR, which was one of the characteristics compared with OLS. 

Some of the information in Figure 4 provides relevant conclusions that are unanimous with the analysis results in Table 5. First, BQR offers empirical results in different quantiles and the upper-lower estimated values, which is one of the characteristics compared with OLS. Second, the effect of INCOME on HCE has a high degree of stability in all quantiles, which implies that both high- and low-income individuals attach great importance to HCE. Third, the regression coefficient of CO_2_ increases gradually as the quantile value increases, especially after 0.5 quantile. In the 0.1 quantile, HCE increases by 0.101 for every 1 unit increased of CO_2_ (as shown in Table 5). In the quantile of 0.9, HCE increases by 0.227 for every 1 unit increased of CO_2_. This indicates that CO_2_ has an increasing role on HCE from the low level to the high level, in spite of HCE at the lower level being little affected by CO_2_.

### 3.5. Time-Trend Analysis

Next, we took data from 2005, 2010, and 2015 as examples to reveal the time evolution of various factors affecting HCE, especially CO_2_. The descriptive statistics of all variables are shown in Table 7.

Table 7 shows a summary of the characteristics of all variables (after log processing) in 2005, 2010, and 2015. Mean HCE keeps increasing (5.87 in 2005, 6.38 in 2010, and 7.1 in 2015), and is synchronized with INCOME and CO_2_. Visual test for MCMC convergence is omitted here since it has been done before.

Table 8 shows the parameter estimation of Bayesian quantile regression for the whole series in 2005, 2010, and 2015, revealing interesting findings.

First, the influence of INCOME on HCE gradually decreases, year by year. For example, the estimated coefficients of INCOME were 0.328 in 2005, 0.234 in 2010, and approximately 0 in 2015 (τ = 0.5), respectively, which implies that people are paying more attention to their own health and are willing to pay a fixed amount for HCE.

Second, the effect of CO_2_ on HCE increases and takes the shape of an inverted U, similar to the Environmental Kuznets Curve (EKC). For instance, the estimated coefficients of CO_2_ were 0.005 in 2005, 0.155 in 2010, and 0.106 in 2015 (τ = 0.5), respectively. 

Third, the change in DCLI is the most significant, compared with other variables, except INCOME and CO_2_. DCLI and HCE grow together, which implies that people are focusing on both short-term health and long-term prevention. 

**Figure 5:**
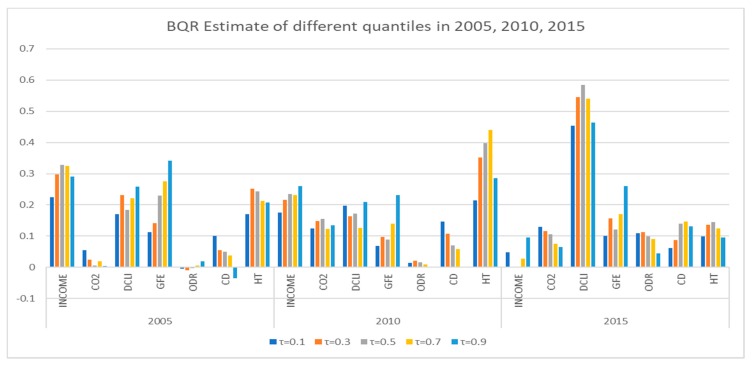
Estimated results of BQR at different quantiles in 2005, 2010, and 2015.

Similar results on the effect of CO_2_ on HCE can be obtained from Figure 5. First, both CO_2_ and DCLI have a more significant positive impact on HCE in all quantiles. Second, CO_2_ had an increasing influence on HCE during 2005–2015. Figure 5 shows that CO_2_ essentially had no effect on HCE in 2005, but CO_2_ had a significant impact on HCE in 2010 and 2015. Finally, there is a heterogeneity in the influence of CO_2_ on HCE in different quantiles, and it becomes more obvious with time. For example, the regression coefficient of CO_2_ decreases gradually as the quantile value increases in 2015, which means that the influence of CO_2_ on HCE at high quantiles is inferior to that at low quantiles.

## 4. Discussion 

Although the role of air pollution in health care expenditure has attracted the attention of scholars in China, little research uses the BQR method (which has been studied in depth theoretically and in biomedical applications) in this field, especially using macro data. Like our last research paper [34], this paper used Bayesian quantile regression to explore the impact of air pollution (such as CO_2_) on healthcare expenditure (HCE). We replaced IWGE (Industrial Waste Gas Emission) with CO_2_ when all other variables were maintained the same, which is because CO_2_, as the most important part of IWGE in China, was adopted in most relevant literature, and can be accepted by most scholars. In fact, some of our conclusions are similar to that of our previous paper. However, our research view is different. Unlike the last paper [34], which focused on a comparison between regions, this paper considered the whole country. Furthermore, we included research on the trend of time evolution. Other important and interesting phenomena were also revealed.

CO_2_ is an important factor affecting healthcare expenditure (HCE) in China, identified by the coefficients of CO_2_ on HCE in all quantiles. The coefficients of CO_2_ were 0.101 (τ = 0.1), 0.157 (τ = 0.5), and it increased to 0.227 (τ = 0.9), which revealed that CO_2_ emissions have an increasing influence on HCE. However, this effect was less compared to INCOME, which showed that the majority of people are not paying sufficient attention to the correlation between air pollution and healthcare [34]. In addition, we also found that income and government financial expenditure were the most important factors affecting HCE for all people, similar to findings in other literature [41]. Thus, the government should focus on environmental control and the increasing investment needs to promote environmental protection, increase technology transfer, and mitigate environmental damage [17]. For example, promoting cleaner production and comprehensive utilization of resources in industrial production.

The influence of CO_2_ on HCE has two specific characteristics: First, from a national perspective, the regression coefficient of CO_2_ increases gradually as the quantile value increases, especially after 0.5 quantile. As is shown in Table 5 and Figure 3, HCE increases by 0.101 for every 1 unit increase of CO_2_ in the 0.1 quantile; it was 0.227 for every 1 unit increase of CO_2_ in the 0.9 quantile. This indicates that CO_2_ plays an increasing role in HCE from low to high levels, in spite of HCE at a lower level being less affected by CO_2_. This conclusion reveals that people in China have obviously different understandings of the correlation between air pollution and healthcare. To be specific, people at low quantiles were more inclined to ignore the hazards of air pollution and not have adequate measures in place for early prevention of health problems (such as various chronic diseases). This also reveals that the majority of people do not pay sufficient attention to the correlation between air pollution and healthcare, in spite of a slow transition. Therefore, efforts by the government—media exposure and increasing publicity—to raise civic awareness of air pollution control and disease prevention is obviously very important [20,34]. In addition, the government should also monitor ambient air pollution levels and disclose to the public information on air quality, related health risks, and strategies to reduce exposure, in a manner that is easily understood.

Second, from the perspective of time evolution, the effect of CO_2_ on HCE increases overall, but takes on the shape of an inverted U, which is similar to the Environmental Kuznets Curve (EKC). For example, CO_2_ had essentially no effect on HCE in 2005, but a significant impact in 2010 and 2015; the estimated coefficients of CO_2_ were 0.005 in 2005, 0.155 in 2010, and 0.106 in 2015 (τ = 0.5), respectively. This indicates that people are indeed paying more attention to environmental influences on physical health; however, it will show a relatively stable state after a certain stage, such as the peak of EKC. This conclusion is different from that in other literature, but similar to that of Hao and others [14], who mentioned that certain indicators of social development and public services deserve more attention after a certain period to mitigate the adverse effect of environmental pollution on health expenditure. Therefore, the government can plan and rationally allocate and distribute healthcare resources to improve public services and reduce the effect of environmental pollution on HCE.

Finally, the applicability of the BQR approach, as an attempt to expand existing literature on the topic, was identified by a preliminary comparison with traditional empirical tools, such as BLR, QR, and OLS. We conclude that the BQR method is reasonably practicable in this research field, since it exhibits several unique characteristics, when compared with other traditional empirical approaches (as shown in Table 6 and Figure 3). Although there is a slight difference (which may be related to insufficient sample size), the results of BQR and other methods are close and the difference—in basic theoretical principles of the Bayes method and the frequency method—is getting smaller. Our research focuses on its applicability rather than its superiority, in a bid to expand and deepen the research area.

Although the theoretical and practical applications of the BQR method have been studied in depth in many fields, there is room for development in the research area discussed in this paper. The prominent problems identified are to do with conversion of data attributes, sample size selection, and measuring the posterior density function. In future research, we will consider more value-independent variables, such as SO_2_, family status, education level, and differences between urban and rural areas, among others.

## 5. Conclusions

The contribution of this paper lies in its attempt to use the Bayesian quantile regression model (BQR) as a newish empirical tool to discuss the correlation between CO_2_ emission and healthcare expenditure (HCE) in China; this is a continuation of our previous work. Admittedly, there are several undeveloped aspects of this topic that need to be addressed in future research. We are not trying to prove superiority of the model, but emphasize its applicability in an effort to expand and deepen research discussions on this topic.

## Figures and Tables

**Table 1:** A summary of related studies.

Reference	Object	Methodology	Dependent Variables	Independent Variables	Main Conclusion
Chaabouni, S; Saidi, K (2017)	51 countries	Generalized method of moments (GMM)	Health spending (HS)	CO_2_ emissions (C), GDP per capita (Y), stock capital (K), population ageing (POP), urbanization (URB) and trade openness (TO)	There is a bidirectional causality between CO_2_ emissions and GDP per capita, between health spending and economic growth for the three groups of estimates.
Khoshnevis Yazdi, S., & Khanalizadeh, B (2017)	MENA countries	Autoregressive Distributed Lag (ARDL) method	Health expenditure	GDP, CO_2_ emissions and PM10	Income and CO_2_ and PM10 emissions have statistically significant positive effects on health expenditure
Usman M, Ma Z, Wasif Zafar M, Haseeb A, Ashraf RU (2019)	13 emerging economies	Lagrange Multiplier (LM) bootstrap approach	Government health expenditure, private health expenditure	CO_2_, SO_2_, NOx, GDP, foreign direct investment, population aging, education	CO_2_ emissions and the environment index have a positive and significant influence on government health expenditures
Yahaya, A., Nor, N. M., Habibullah, M. S., Ghani, J. A., & Noor, Z. M (2016)	125 developing countries	Panel co-integration	Health expenditures	Income, NOx, CO_2_, SO_2_, CO	CO_2_ has the highest explanatory power on the per capita health expenditure.
Tian, F.; Gao, J.; Yang, K (2016)	28 OECD countries	Quantile regression approach	Health expenditures	The number of physicians, the proportion of the population aged 65 years and older, GDP, the percent of urban population.	The determinants of per capita healthcare expenditure growth, involving the growth of lagged health spending, per capita gross domestic product (GDP), physician density.
Apergis, N.; Gupta, R.; Lau, C.K.M.; Mukherjee, Z. (2018)	Across U.S. states	Quantile regression	Healthcare expenditures	Personal disposable income per capita, and CO_2_ emissions	The effect of CO_2_ emissions was stronger at the upper-end of the conditional distribution of healthcare expenditures
Chaabouni, S.; Zghidi, N.; Mbarek, M.B (2016)	51 countries	Dynamic simultaneous equations models	GDP, CO_2_ emissionshealth expenditures	Aging population, urbanization, labor employed in production, stock capital, trade openness	There is bidirectional causality between CO_2_ emissions and economic growth, between health expenditures and economic growth for the global panel

**Table 2:** Variable selection and definition.

Variable Types		Variable Name	Variable Definition
Dependent Variable		ln *HCE*	Per capita health expenditure (yuan) in terms of natural logarithms
Independent Variables	(a) Environment pollution variables	ln *CO_2_*	Per capita carbon dioxide (CO_2_) emissions (ton/10,000 people) in terms of natural logarithms
	(b) Economic variables	ln *INCOME*	Per capita income (yuan) in terms of natural logarithms
		ln*GFE*	Per capita government financial expenditure (yuan) in terms of natural logarithms
		ln*DCLI*	Density of commercial life insurance in terms of natural logarithms
	(c) Public service variables	ln*HT*	Number of health technicians per thousand population in terms of natural logarithms
	(d) Family and personal variables	ln*ODR*	Old dependency ratio in terms of natural logarithms
		ln*CD*	The scale of chronic disease (1000 people) in terms of natural logarithms

Notes: HCE: Health care expenditure, CO2: Carbon dioxide, INCOME: Income, GFE: Government financial expenditure, DCLI: Density of commercial life insurance, ODR: Old dependency ratio, CD: Chronic disease, HT: health technician.

**Table 3:** Summary of statistics for all the variables.

Variables	Mean	SD	Median	Trimmed	Skew	Kurtosis	Mean	SD	Median	Trimmed	Skew	Kurtosis
	Part A: In Original Value						Part B: In Log Difference					
HCE	688.32	385.15	613.23	644.47	1.09	1.34	6.38	0.57	6.42	6.39	−0.13	−0.61
INCOME	13,174.28	7797.48	11,526.37	12,091.22	1.48	2.89	9.33	0.56	9.35	9.32	0.06	−0.63
CD	1082.22	650.26	936.29	1034.84	0.59	−0.52	6.76	0.75	6.84	6.84	−0.82	0.16
CO_2_	4.19	3.04	3.28	3.68	2.26	8.36	1.22	0.64	1.19	1.21	0.2	−0.29
GFE	0.66	0.48	0.56	0.59	1.36	2.09	−0.68	0.76	−0.58	−0.67	−0.18	−0.79
DCLI	696.79	764.38	497.27	533.73	3.04	10.07	6.18	0.83	6.21	6.16	0.25	0.25
ODR	12.52	2.41	12.36	12.37	0.54	0.02	2.51	0.19	2.51	2.51	0.09	−0.47
HT	4.82	1.91	4.48	4.56	2.07	6.64	1.51	0.35	1.5	1.5	0.48	0.77

**Table 4:** Results of panel stability test—augmented Dickey-Fuller test (ADF).

Variable	Dickey-Fuller	Variable	Dickey-Fuller	Variable	Dickey-Fuller
HCE	−8.638 ***	GFE	−9.062 ***	CD	−3.186 *
INCOME	−8.827 ***	ODR	−4.293 ***	HT	−6.409 ***
CO_2_	−6.147 ***	DCLI	−7.161 ***	——	——
Pool-test (effect=individual)	F: 0.2644 (*p*-value: 0.9589)		Pool-test (effect=time)	F: 0.7186 (*p*-value: 0.9566)	

Note: “*” and “***” represent *p*-value < 0.10 and *p*-value < 0.01, respectively.

**Table 5:** Coefficient estimates of BQR in different quantiles (τ = quantile).

Variables/Quantiles	τ = 0.1	τ = 0.3	τ = 0.5	τ = 0.7	τ = 0.9
INCOME	0.356 **	0.344 **	0.331 **	0.271 **	0.313 **
CD	0.092 **	0.096 **	0.083 **	0.066 **	0.023 **
CO_2_	0.101 **	0.104 **	0.157 **	0.215 **	0.227 **
GFE	0.302 **	0.278 **	0.274 **	0.316 **	0.312 **
DCLI	–0.001 **	0.078 **	0.089 **	0.072 **	0.051 **
ODR	0.188 **	0.087 **	0.025 **	0.018 **	–0.008 **
HT	0.262 **	0.249 **	0.227 **	0.221 **	0.183 **

Note: The results were 95% credible interval in BQR, which has the same meaning as *p* < 0.05 in OLS, thus, ** were shown. The number of retained draws and burn in draws were 4000 and 1000 each.

**Table 6:** Estimation results of Bayesian quantile regression (BQR) and traditional empirical methods (tau = 0.5/mean).

Variables	OLS	QR	BLR	BQR
INCOME	0.337 *** (0.054)	0.348 ** (0.049)	0.336 *** (0.054)	0.331 ** (0.099)
CD	0.076 ** (0.024)	0.017 ** (0.022)	0.076 *** (0.024)	0.083 ** (0.05)
CO_2_	0.118 *** (0.022)	0.104 ** (0.015)	0.085 *** (0.022)	0.157 ** (0.04)
GFE	0.317 *** (0.046)	0.171 ** (0.028)	0.316 *** (0.046)	0.274 ** (0.083)
DCLI	0.080 * (0.047)	0.118 ** (0.022)	0.102 *** (0.047)	0.089 ** (0.092)
ODR	0.084 * (0.02)	0.233 ** (0.039)	0.045 *** (0.02)	0.025 ** (0.036)
HT	0.215 *** (0.037)	0.353 ** (0.084)	0.216 *** (0.037)	0.227 ** (0.076)

Note. “*”, “**”and “***” represent *p*-value < 0.10, *p*-value < 0.05 and *p*-value < 0.01, respectively. The results of both QR and BQR showed 95% credible interval (that is, “**”). In BQR, the number of retained draws and burn in draws were 4000 and 1000 each.

**Table 7:** Descriptive statistics of all variables (after log processing).

Variables	Mean	SD	Skew	Kurtosis	Mean	SD	Skew	Kurtosis	Mean	SD	Skew	Kurtosis
	2005				2010				2015			
HCE	5.87	0.44	0.83	0.32	6.38	0.35	0.23	−0.76	7.1	0.29	0.07	−0.16
INCOME	8.74	0.41	1.05	0.16	9.39	0.37	0.93	−0.02	9.98	0.3	1.23	0.76
CD	6.72	0.78	−0.77	−0.2	6.76	0.76	−0.79	0.02	6.8	0.74	−0.79	0.12
CO_2_	0.7	0.55	0.37	−1.25	1.35	0.6	1.21	1.34	1.56	0.54	0.2	−0.69
GFE	−1.58	0.48	1.27	1.36	−0.5	0.38	0.87	−0.25	0.18	0.36	0.84	−0.31
DCLI	5.45	0.8	1.35	2.12	6.39	0.66	1.12	1.97	6.93	0.54	0.62	1.03
ODR	2.5	0.18	−0.21	−1.02	2.44	0.17	0.2	−0.78	2.61	0.18	−0.3	−0.69
HT	1.31	0.34	0.94	0.9	1.52	0.34	1.13	1.83	1.78	0.16	1.27	3.2

**Table 8:** Quantile regression estimation of different quantiles in 2005, 2010, and 2015.

Year	Bayes Estimated/Quantile	τ = 0.1	τ = 0.3	τ = 0.5	τ = 0.7	τ = 0.9
2005	**INCOME**	0.225 **	0.298 **	0.328 **	0.324 **	0.291 **
	**CD**	0.100 **	0.055 **	0.050 **	0.038 **	−0.035 **
	**CO_2_**	0.055 **	0.024 **	0.005 **	0.019 **	0.004 **
	**GFE**	0.113 **	0.141 **	0.229 **	0.275 **	0.342 **
	**DCLI**	0.171 **	0.232 **	0.184 **	0.222 **	0.258 **
	**ODR**	−0.005 **	−0.010 **	−0.003 **	0.005 **	0.019 **
	**HT**	0.171 **	0.252 **	0.244 **	0.212 **	0.207 **
2010	**INCOME**	0.175 **	0.216 **	0.234 **	0.232 **	0.260 **
	**CD**	0.146 **	0.107 **	0.070 **	0.058 **	0.003 **
	**CO_2_**	0.124 **	0.148 **	0.155 **	0.123 **	0.135 **
	**GFE**	0.069 **	0.098 **	0.088 **	0.139 **	0.232 **
	**DCLI**	0.197 **	0.163 **	0.172 **	0.127 **	0.209 **
	**ODR**	0.014 **	0.021 **	0.016 **	0.009 **	−0.001 **
	**HT**	0.215 **	0.352 **	0.398 **	0.441 **	0.285 **
2015	**INCOME**	0.048 **	0.001 **	−0.002 **	0.027 **	0.096 **
	**CD**	0.061 **	0.087 **	0.140 **	0.147 **	0.131 **
	**CO_2_**	0.130 **	0.116 **	0.106 **	0.076 **	0.065 **
	**GFE**	0.101 **	0.156 **	0.121 **	0.170 **	0.260 **
	**DCLI**	0.453 **	0.545 **	0.584 **	0.541 **	0.464 **
	**ODR**	0.110 **	0.112 **	0.099 **	0.090 **	0.044 **
	**HT**	0.099 **	0.136 **	0.144 **	0.125 **	0.095 **

Note: All of the outcomes are at 95% credible interval. Number of burn-in draws: 1000, number of retained draws: 4000.

## Abbreviations

CO_2_: Carbon dioxide; HCE: healthcare expenditure; BQR: Bayesian quantile regression; OLS: ordinary least squares; QR: quantile regression; BLR: Bayesian linear regression; MCMC: Markov Chain Monte Carlo.
